# Unexpected Right Atrial Crossing of the Sphere‐9 Catheter Despite Stable Left Atrial Sheath Position

**DOI:** 10.1002/joa3.70462

**Published:** 2026-09-21

**Authors:** Takeru Ikenaga, Masatsugu Nozoe, Takanori Watanabe, Hiroshi Mannoji, Toru Kubota

**Affiliations:** ^1^ Division of Cardiology, Cardiovascular and Aortic Center Saiseikai Fukuoka General Hospital Fukuoka Japan

**Keywords:** Affera Sphere‐9, atrial fibrillation, high density mapping, patent foramen ovale, residual transseptal puncture site, unexpected right atrial crossing

## Abstract

The Sphere‐9 catheter unexpectedly crossed into the right atrium despite stable left atrial sheath position. A perpendicular, sheath‐supported approach may have facilitated passage of the flexible lattice tip through a small interatrial communication while the sheath remained within the left atrium.

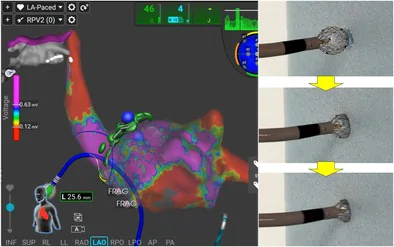

The Affera dual‐energy lattice‐tip platform enables both mapping and ablation with a single Sphere‐9 catheter [1, 2, 3, 4], but its unique design may also create previously unrecognized procedural pitfalls. A 72‐year‐old man with a history of radiofrequency ablation using two left atrial (LA) sheaths 2 years earlier underwent repeat ablation for recurrent atrial fibrillation with the Affera Sphere‐9 system. An initial LA voltage map suggested that the left superior, left inferior, and right inferior pulmonary veins (PVs) remained electrically isolated. Additional detailed mapping around the right superior PV and LA septum was performed with the Sphere‐9 catheter in a deflected‐back configuration through a steerable sheath. During this mapping, the catheter suddenly advanced into an unexpected chamber with recordable voltage (Figure 1). Simple withdrawal of the catheter allowed it to return to the LA without any obvious change in sheath position (Figure 2). Because the sheath position appeared unchanged within the LA, the unexpected space was initially interpreted as a reconnected right superior PV, and pulsed field ablation (PFA) was therefore delivered. During PFA application around the right superior PV, the catheter was manipulated directly through the steerable sheath without a deflected‐back configuration, and the same phenomenon did not occur. The phenomenon occurred reproducibly only when the catheter was deflected back during mapping of the LA septum and was not observed without this maneuver (Figure 3A–C). Subsequent post‐ablation mapping of the LA revealed that the unexpected space was not a reconnected right superior PV, but rather a right atrial (RA) chamber continuous with the superior vena cava (Figure 3D–F). These findings supported unexpected RA crossing of the Sphere‐9 catheter presumably through a patent foramen ovale or residual transseptal puncture site. During PFA delivery, the catheter did not enter the unexpected chamber; therefore, no inadvertent PFA was applied within the RA (Figure 3D).

**FIGURE 1:**
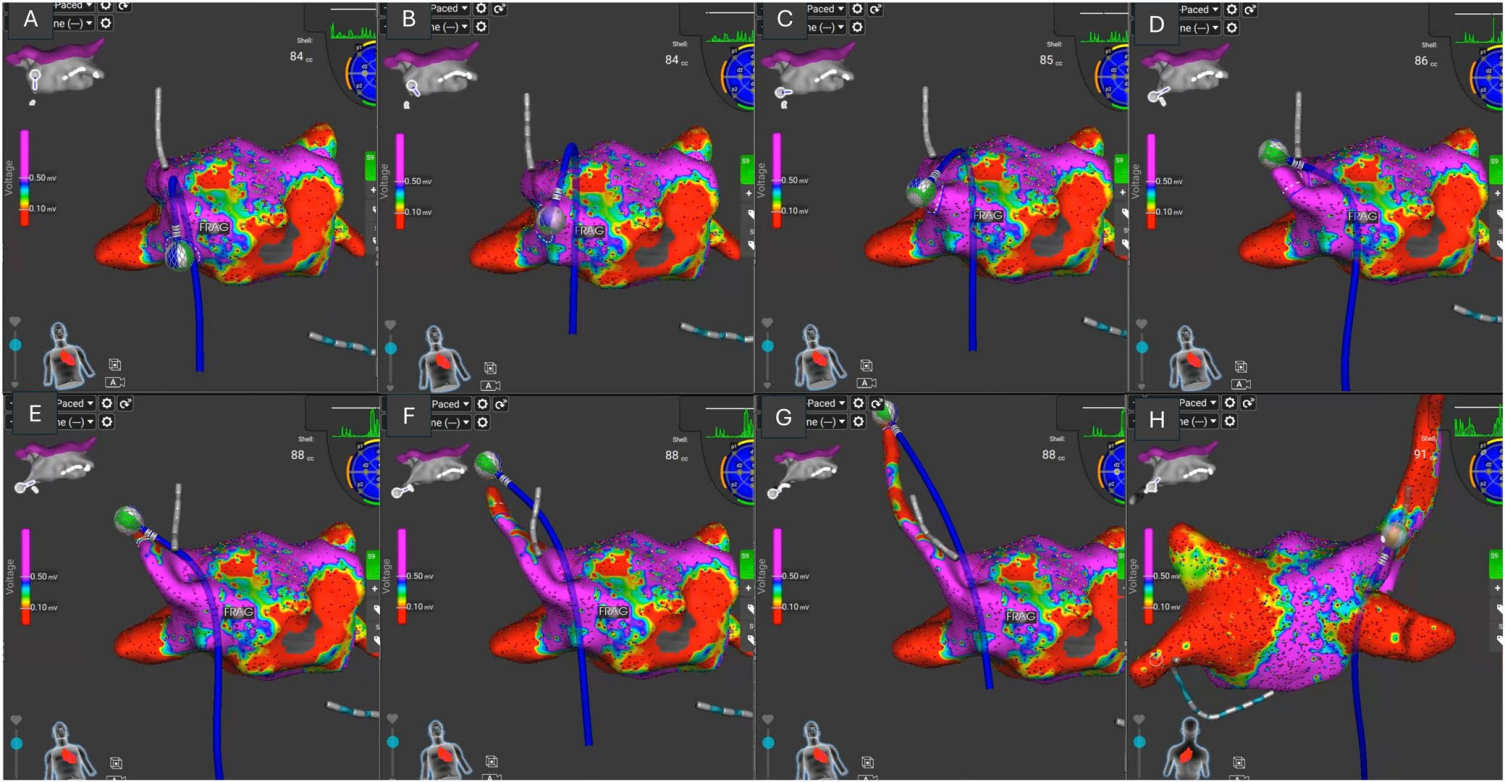
Unexpected right atrial crossing of the Sphere‐9 catheter during left atrial septal mapping. (A, B) Mapping of the left atrial (LA) septum was performed with the Sphere‐9 catheter in a deflected‐back configuration using a steerable sheath. (C–H) The catheter suddenly advanced into an unexpected space with recordable voltage. This space was initially interpreted as a reconnected right superior pulmonary vein.

**FIGURE 2:**
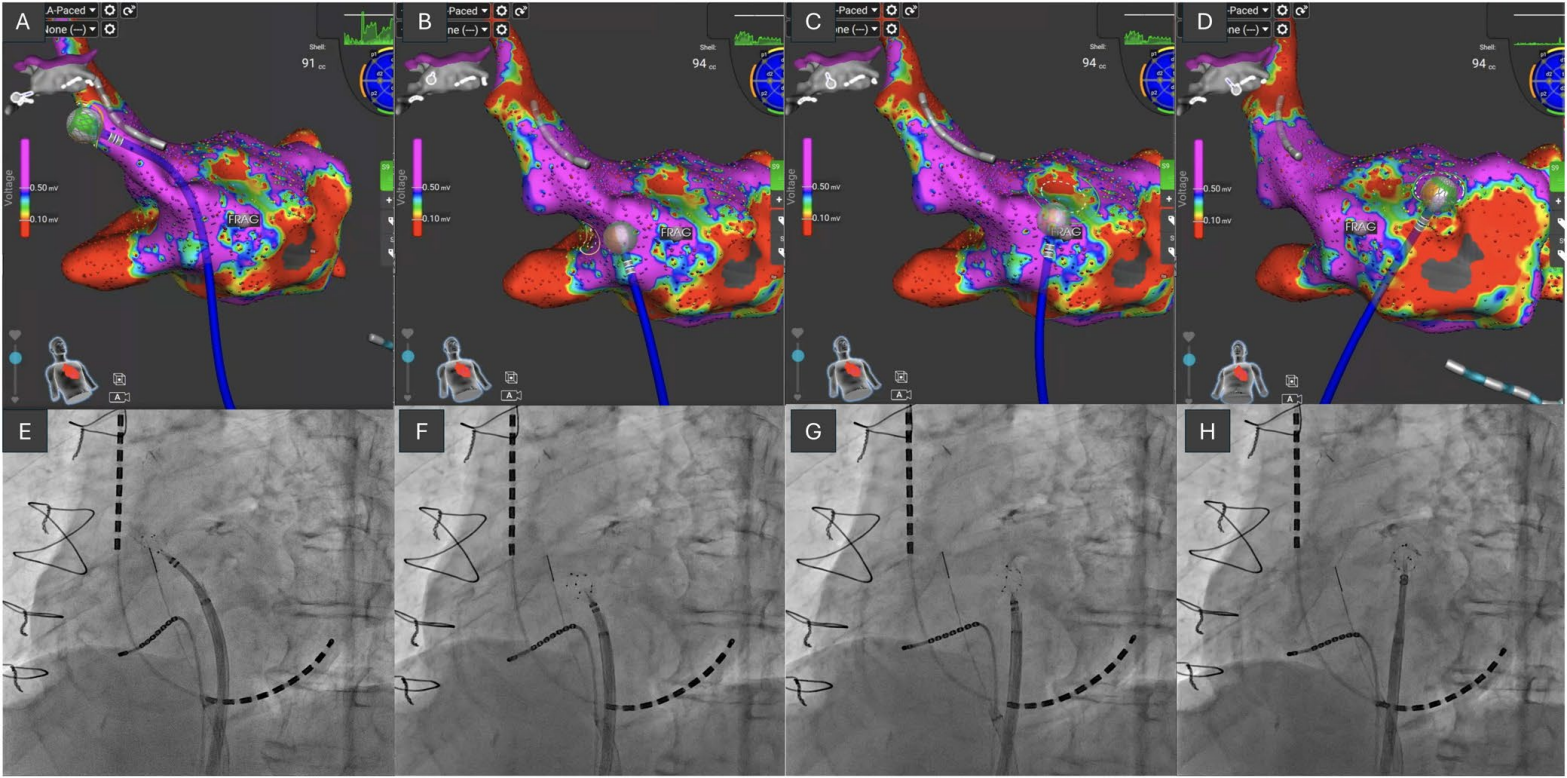
Stable left atrial sheath position during right atrial crossing of the Sphere‐9 catheter. (A) Voltage mapping using Affera showed the position of the Sphere‐9 catheter as it suddenly advanced into an unexpected space with recordable voltage. (B–D) Simple withdrawal of the catheter allowed it to re‐enter the left atrium without any obvious change in sheath position. (E–H) Corresponding fluoroscopic left anterior oblique (LAO) views for panels A–D, showing that the sheath remained within the left atrium during these catheter manipulations.

**FIGURE 3:**
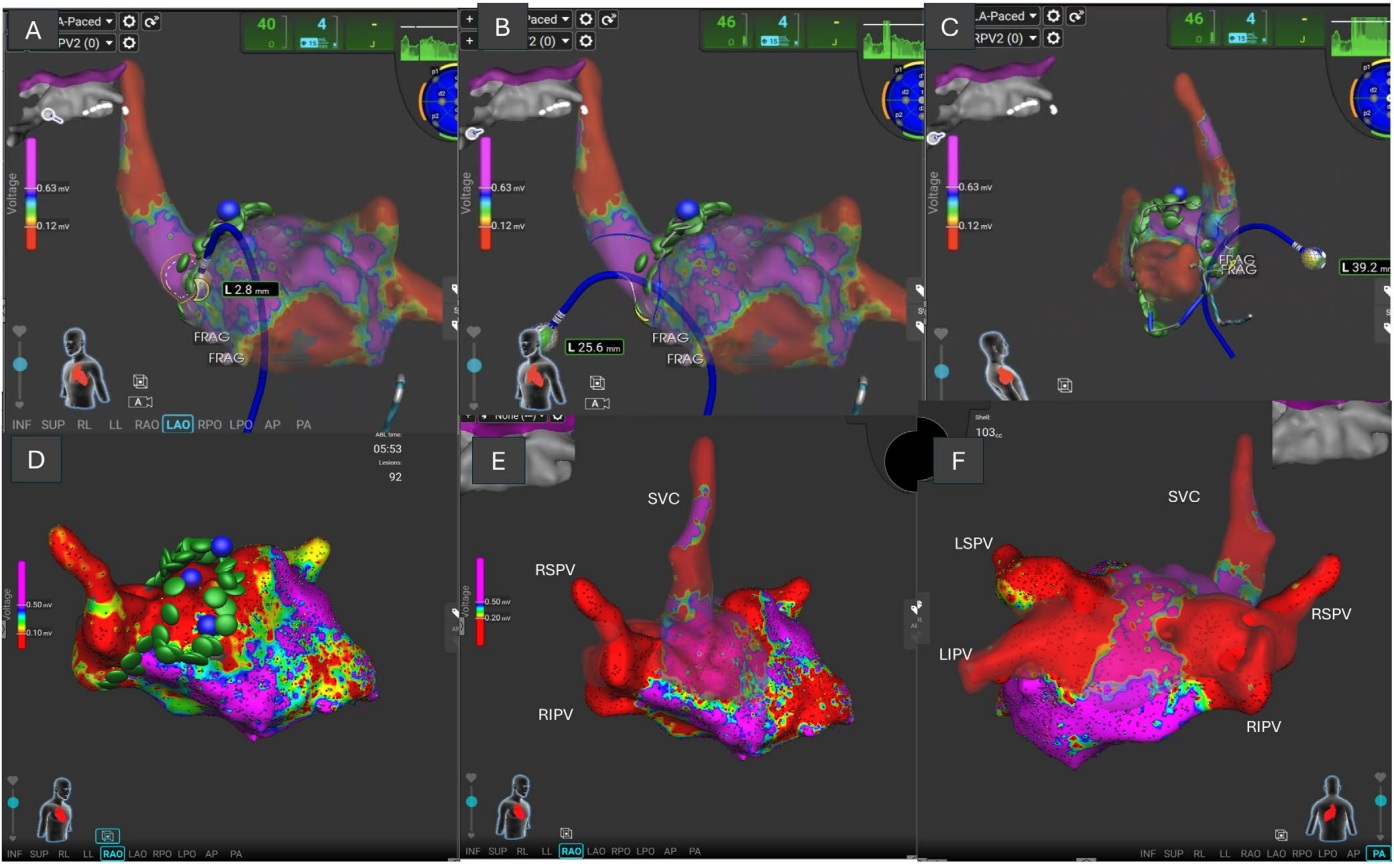
Repeated occurrence of right atrial crossing of the Sphere‐9 catheter. (A) Post‐ablation mapping of the left atrial (LA) septum was performed with the Sphere‐9 catheter in a deflected‐back configuration using a steerable sheath. (B, C) The catheter again suddenly advanced into an unexpected space with preserved voltage. (D) During pulsed‐field ablation (PFA) delivery, the catheter did not enter the unexpected chamber; therefore, no inadvertent PFA was applied within the right atrium. (E, F) Overlaying the LA post‐ablation map with a semi‐transparent rendering of the pre‐ablation map clearly demonstrated that the SVC had been misinterpreted as the right superior pulmonary vein. Right superior pulmonary vein (RSPV), right inferior pulmonary vein (RIPV), left superior pulmonary vein (LSPV), left inferior pulmonary vein (LIPV), superior vena cava (SVC).

Although a similar phenomenon could theoretically occur with other mapping catheters in the presence of an interatrial communication, conventional mapping catheters generally return to the RA when the sheath itself is displaced back into the RA. In contrast, in the present case, only the Sphere‐9 catheter crossed into the RA while the sheath remained within the LA, and simple catheter withdrawal allowed the catheter to return to the LA, making the catheter position difficult to recognize. During septal mapping in the deflected‐back configuration with the steerable sheath, the flexible lattice tip was pressed nearly perpendicular to the interatrial septum. In a bench experiment, a perpendicular approach with the steerable sheath allowed the Sphere‐9 catheter to pass through a hole smaller than the lattice‐tip diameter with only mild narrowing of the lattice tip, whereas passage did not occur with oblique sheath pressure or without sheath support (Figure 4). These findings suggest that a perpendicular, sheath‐supported approach may have facilitated passage of the flexible lattice tip through a small interatrial communication while the sheath remained within the LA. Because this phenomenon may lead to misinterpretation of the catheter position and unnecessary ablation, physicians using the Affera platform should be aware of this potential catheter‐related pitfall, particularly during septal mapping with the catheter in a deflected‐back configuration.

**FIGURE 4:**
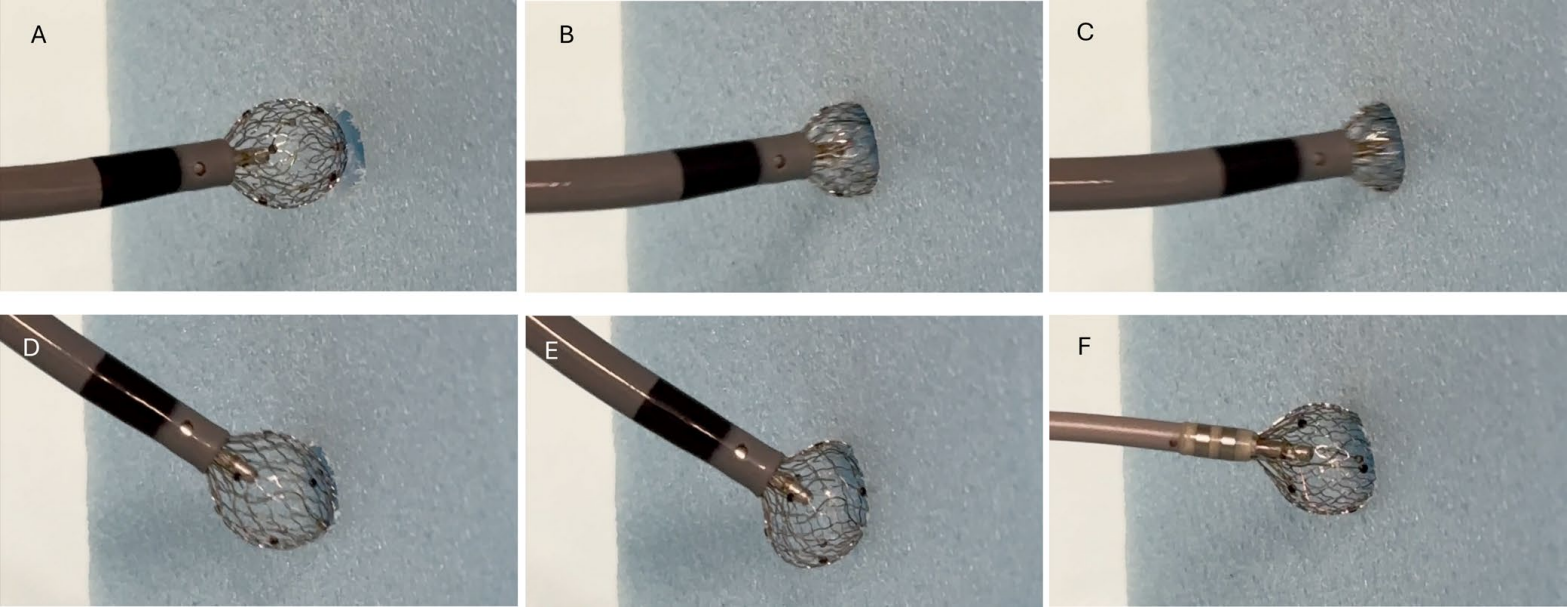
Bench experiment assessing passage of the Sphere‐9 catheter through a hole smaller than the lattice‐tip diameter. (A–C) When the steerable sheath was positioned perpendicular to the hole and advanced toward it, the Sphere‐9 catheter passed through the 6‐mm hole with mild narrowing of the lattice tip but without marked deformation. (D, E) When the sheath was forcefully pressed against the hole at an oblique angle, the lattice tip was deformed, and the catheter could not pass through the hole. (F) When the catheter alone was advanced without sheath support, the catheter shaft bent under the applied force, preventing the lattice tip from passing through the hole.

The precise route by which the Sphere‐9 catheter crossed into the RA could not be determined. Neither preprocedural computed tomography nor transesophageal echocardiography was performed in this case. Although intracardiac echocardiography was used to guide transseptal puncture during the procedure, no dedicated assessment was performed for a patent foramen ovale or a residual interatrial communication related to the previous transseptal puncture. Because the apparent crossing site was clearly distinct from the site of the current transseptal puncture, a patent foramen ovale or a residual defect from the prior procedure was considered a possible route; however, this could not be confirmed.

## Funding

The authors have nothing to report.

## Consent

Written informed consent was obtained from the patient for publication of this case report and any accompanying images in accordance with COPE guidelines.

## Conflicts of Interest

Dr. Masatsugu Nozoe and Dr. Hiroshi Mannoji report receiving speaker fees from Medtronic. The other authors declare no conflicts of interest.

## Data Availability

Data sharing not applicable to this article as no datasets were generated or analysed during the current study.
